# Investigation of common risk factors between polycystic ovary syndrome and Alzheimer’s disease: a narrative review

**DOI:** 10.1186/s12978-021-01203-x

**Published:** 2021-07-26

**Authors:** Nahid Sarahian, Hosna Sarvazad, Elham Sajadi, Nasrin Rahnejat, Narges Eskandari Roozbahani

**Affiliations:** 1 Neuroscience Research Center, Shahid Beheshti University of Medical Sciences, Tehran, Iran; 2 Clinical Research Development Center, Imam Reza Hospital, Kermanshah University of Medical Sciences, Kermanshah, Iran; 3 Department of Basic Science, School of Veterinary Medicine, Shiraz University, Shiraz, Iran; 4 Faculty of Nursing and Midwifery, Kermanshah University of Medical Sciences, Kermanshah, Iran

**Keywords:** Polycystic ovary syndrome, Alzheimer disease, Hyperandrogenism, Insulin resistance, Review

## Abstract

**Background:**

The most common endocrine and metabolic disorders in premenopausal women is polycystic ovary syndrome (PCOS), characterized by hyperandrogenism, chronic anovulation, and/or ultrasound evidence of small ovarian cysts. Obesity and insulin resistance are also the main factors influencing the clinical manifestations of this syndrome. Alzheimer’s disease (AD) is the most typical progressive neurodegenerative disorder of the brain, and recent studies suggest a relationship between endocrinal dysregulation and neuronal loss during AD pathology.

**Aim:**

This study aimed to evaluate the common risk factors for Alzheimer’s and PCOS based on previous studies. Knowing the common risk factors and eliminating them may prevent neurodegenerative Alzheimer’s disease in the future.

**Method:**

In this narrative review, international databases, including Google Scholar, Scopus, PubMed, and the Web of Science, were searched to retrieve the relevant studies. The relevant studies’ summaries were categorized to discuss the possible pathways that may explain the association between Alzheimer’s and PCOS signs/symptoms and complications.

**Results:**

According to our research, the factors involved in Alzheimer’s and PCOS disorders may share some common risk factors. In patients with PCOS, increased LH to FSH ratio, decreased vitamin D, insulin resistance, and obesity are some of the most important factors that may increase the risk of Alzheimer’s disease.

## Plain English summary

Polycystic ovary syndrome is a disorder of the female reproductive system that can be caused by hormonal disorders. The disease is detected by an ultrasound of the ovaries with small ovarian cysts. Obesity and insulin resistance are among the factors that can affect the clinical symptoms of this disease. Obesity due to high-fat consumption can affect cognitive functions with age. Alzheimer’s is the most common disease associated with disorders in brain cells; a link between hormonal disorders and Alzheimer’s has recently been reported. We conducted a review of reports and articles published in connection with polycystic ovary syndrome and neurodegenerative disorders in reputable scientific databases. Studies have shown that the factors involved in polycystic ovary syndrome and Alzheimer’s disease may indicate that both diseases have common risk factors. It may be linked to the symptoms and/or complications of Alzheimer’s disease and polycystic ovary syndrome. Future preclinical studies are needed to closely examine the mechanisms associated with polycystic ovary syndrome and the association with Alzheimer’s. The novelty of our study is from the fact that the PCOS may be to some extent considered as a cause (exposure) among others of AD’s (outcome) and the association might be confounded by some or all the risk factors assessed in this review. The nature of the method—the narrative review—is relatively subjective (in the determination of which studies to include, the way the studies are analyzed, and the conclusions drawn) and hence may not help mitigate bias.

## Introduction

Polycystic ovary syndrome (PCOS) is a heterogeneous group of disorders with metabolic and endocrine disorders that affect 8–10% of women of reproductive age [1]. PCOS often shows a wide range of metabolic changes, such as insulin resistance (IR), blood lipids, obesity, inflammation, increased oxidative stress, and hormonal changes [2]. The exact cause of PCOS is not known, but genetic and environmental factors appear to be involved. Recent studies have shown that hyperandrogenism (HA) and IR are the main causes and primary features of PCOS. Furthermore, these factors can influence the onset and progression of PCOS [3].

IR directly or indirectly enhances androgen synthesis and secretion; hyperandrogenism stimulates the breakdown of visceral adipose tissue, leading to an increase in free fatty acids, which intensifies IR levels. Eventually, this can create a vicious cycle between hyperandrogenemia and IR in PCOS and lead to development and progression [4]. Insulin also affects central nervous system (CNS) function by playing a role in regulating energy homeostasis, central action on peripheral glucose metabolism, learning, memory, cognition, and survival of brain neurons in adults [5]. Therefore, any disturbance in insulin metabolism in the CNS may have adverse effects on brain function and cognitive activity [6].

Alzheimer’s disease (AD) is an irreversible and progressive disorder with neuronal destruction characterized by behavioral changes and cognitive function loss in the elderly. AD accounts for 60 to 80% of dementia cases [7]. Approximately 46 million people have dementia globally, and this number is projected to increase to 131.5 million by 2050 [8]. Common symptoms include short-term memory loss, cognitive impairment, and inability to perform tasks in daily life [9]. Definitive diagnosis of the disease is usually made only after postmortem examination in which the histopathological examination of senile plaques and neurofibrillary tangles tau can be diagnosed. However, today there are research institutes that can diagnose amyloid and tau burden in living patients, and thus this historical paradigm has challenged the definitive diagnosis of the disease [10].

Intracellular inclusions of tau protein in the form of neurofibrillary tangles and extracellular plaque formation by the accumulation of the amyloid beta-peptide (Aβ) derived from the β-amyloid precursor protein (APP) are two primary pathological lesions in the brains of Alzheimer’s patients [11]. Neurosteroids and sex steroids have been suggested as one reason for reducing the pathology of AD. Alzheimer’s is more common in women than men, and estrogen depletion is generally associated with an increased risk of AD [12]. The age-related decrease in brain levels of testosterone in men and 17β-estradiol (E2) in women during menopause has been associated with a greater risk of developing AD [12].

Loss of neurons and synapses leads to atrophy of the cortex and subcortical areas [13]. Neurodegeneration in AD is associated with unregulated lipid and carbohydrate metabolism, cytokine-mediated inflammation, increased oxidative and cellular stress, ongoing cell death, and vascular destruction [14]. These factors are also present in type 2 diabetes, metabolic syndrome, and non-alcoholic fatty liver disease and support the notion that insulin-resistant diseases are all related and could have the same origins, and may be managed by similar if not identical therapeutic strategies.

Evidence suggests that, in addition to hormonal disorders and obesity, vitamin deficiencies and sleep disorders are also risk factors for this disease [15].

There is no effective treatment to prevent or slow the progression of AD. But in this context, it should also be emphasized that factors considered protective, such as physical exercise, diet, and cognitive stimuli should be strongly and widely encouraged [16].

Because Alzheimer’s patients have risk factors similar to those of PCOS, such as insulin resistance, vitamin D deficiency, sexual hormonal changes, inflammation, and sleep disorders, it may be hypothesized that PCOS may increase the risk of Alzheimer’s disease. This narrative review aimed to summarize the possible pathways that may explain the association between Alzheimer’s and PCOS.

## Methods

**Fig. 1:**
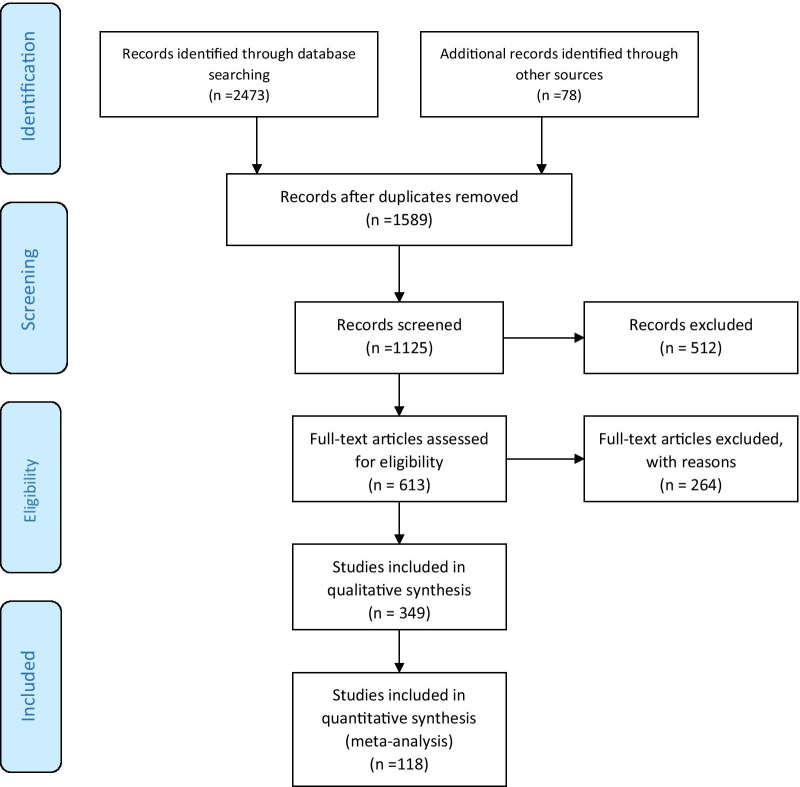
The flow diagram of study selection for the present literature review on the poly cystic ovary syndrome (PCOS), and Alzheimer’s disease

A comprehensive search of Google Scholar, Scopus, PubMed, and the Web of Science (until February 01, 2021) was conducted during this review. Search parameters, MeSH indexing terms, included polycystic ovary syndrome, PCOS, vitamin D, obesity, sex hormones, androgens, estrogens, progesterone, insulin resistance, sleep apnea, memory, Alzheimer disease, and dementia. All original and review studies in English based on the keywords searched in the database mentioned above, studies on humans and animal models, and published from 1973 to 2021 were included in our study. Non-English studies, conference abstracts, chapters of books, letters to the editor, and ex vivo studies were excluded. At the last screening among articles that had met the inclusion criteria, those which repeatedly stated the scientific facts were removed, and those with more up-to-date topics were selected. In addition, in all cases, common pathways leading to Alzheimer’s disease and PCOS were considered. A total of 2876 articles from four databases were identified, and 118 studies that had inclusion criteria were subjected to careful review (Fig. 1).

## Results

### Hypothalamic–pituitary–gonadal axis and estrogen

The hypothalamic–pituitary–gonadal (HPG) plays an essential role in regulating various activities, and an impaired HPG axis can cause polycystic ovary syndrome; sex hormone levels are regulated by the HPG [17, 18]. Deregulation in the HPG axis leads to menopause in women and andropause in men [19].

HPG axis hormone receptors (including steroid hormones, human chorionic gonadotropin, LH, and GnRH) are concentrated in the limbic system, especially hippocampal pyramidal neurons, and are involved in regulating the growth, structure, and function of the adult brain. These sections are very sensitive to AD pathology [20–22].

Estrogen receptors (ERs) by activating brain-derived neurotrophic factor (BDNF) have an essential effect on AD, and a significant reduction in postmenopausal estrogen may increase the risk of Alzheimer’s [23, 24]. Decreased sex gland hormones and increased gonadotropins such as LH are involved in cognitive dysfunction in aging and the pathogenesis of age-related disorders such as AD. This is especially important for women, who are twice as likely to develop postmenopausal AD [25]. The estrogen receptor network is one of the significant regulatory systems in the brain; under its influence, the brain responds to the ovarian-neural estrogen axis at appropriate intervals to regulate its energy metabolism [26]. The Hippocampus, Prefrontal cortex, amygdala, and posterior cingulate cortex of the brain regions with substantial estrogen receptors are essential for learning and memory [27].

Because estrogen’s protective effect on the brain is known, estrogen loss during menopause can partly lead to impaired brain metabolism and mitochondrial dysfunction in AD [28]. In this regard, several studies have shown a strong association between decreased estrogen levels during menopause and AD development [11, 29]. Studies using estradiol as the physiological form of estrogen have also shown that estrogen stabilizes or improves cognition in women with AD [30]. Estrogen modulates neurogenesis by modulating learning and memory in the hippocampus in various species, from rodents to primates [31].

Due to the significant role of hormones in memory and cognition, in addition to clinical and epidemiological studies of estrogen and androgens in AD, it is necessary to study other hormones involved in the HPG axis.

### Hypothalamic–pituitary–gonadal axis dysfunction

The ratio of luteinizing hormone (LH) to follicle-stimulating hormone (FSH) (LH/FSH ratio) in women with PCOS is significantly higher than in normal women of the same age [32], and increasing this ratio is a powerful indicator of PCOS [33]. In these patients, the increase in LH secretion may be due to a defective hypothalamic–pituitary–ovarian axis [34]. LH is a gonadotropin secreted by the anterior pituitary gland that binds to luteinizing hormone receptors (LHR) and is found in the tissues of the gonad and non-gonad [35]. In patients with PCOS, by increasing LH, the ovarian theca cells produce excess androgens, and low FSH contributes to impaired folliculogenesis and ovulation [3]. Interestingly, IR and hyperinsulinemia, which are prominent PCOS features, stimulate LH secretion from the pituitary gland (thus increasing the LH/FSH ratio) and increase androgen production in ovarian follicular cells [36]. LH in the ovaries and testes stimulates the production of gonadal hormones, including estrogens and androgens [37]. An increase in LH increases androstenedione’s production is converted to testosterone by 17β reductase in thecal cells and released into the blood [38]. Androstenedione can also be converted to estradiol by the FSH-dependent aromatase enzyme in ovarian granulosa cells [39].

Because in PCOS, LH levels are high compared to FSH, androstenedione accumulates in the ovaries. In the early stages, androstenedione causes the follicles to grow; however, the persistence of high levels promotes the growth of most follicles as they mature, so ovulation does not lead to the accumulation of antral follicles, and the ovary becomes polycystic [40]. Increased LH levels compared to FSH in PCOS cause more conversion of androstenedione to testosterone, leading to hirsutism [41].

LH increases with age in both men and women. This hormone may be the main hormone in cognitive dysfunction and AD [42]. In this regard, the evidence in epidemiological studies supports the increase of LH in exacerbating the age-related cognitive decline in men and women [42]. There is an inverse relationship between LH and memory, and most studies show a correlation between serum LH concentration and amyloid-beta plasma accumulation and deposition [43]. Elevated LH levels are involved in Aβ metabolism and accumulation and are associated with an increased risk of AD [20]. Some studies suggest a link between LH and BDNF in the hippocampus, and these studies suggest that estrogen-increasing and LH-lowering treatments may both require BDNF signaling to improve spatial memory [44]. While most studies have shown that LH increases BDNF levels in gonadal tissue, some studies have shown that LH decreases BDNF levels in the CNS [44]. Genetic defects in the amyloid beta-AD pathway may increase GnRH and LH, which in turn may have a spiral effect on AD neurotoxicity.

There is not much research on the possible effects of FSH in this area, although one study found that there was a correlation between higher cognitive function in older women and increased FSH levels [45]. However, as mentioned earlier, LH has the most significant effect on the hippocampus, and more research is needed.

### Insulin resistance (IR)

Insulin resistance is a pathological condition in which cells fail to respond to normal circulating insulin levels, so insulin cannot provide average glucose and lipid homeostasis [46]. IR and hyperinsulinemia play a major role in the cause of PCOS, and 50% to 70% of these patients are presented with IR [47]. IR is an essential regulator of androgen synthesis and IR resistance is common in some diseases, such as PCOS, AD, Parkinson’s diseases, and several cancers [3, 48–50].

Insulin is a major trophic factor in brain development [51] and, insulin irregularity and IR are other features of endocrine changes in the pathology of AD that may play a role in disease progression [52]. As a result, patients with impaired insulin metabolism may be at a higher risk of developing Alzheimer’s disease [5]. Women with PCOS are more prone to a wide range of complications from metabolic disorders, so the question is whether PCOS can make women more susceptible to AD?

Metabolic diseases impair brain health and cognitive function [53], and insulin signaling affects the hippocampus’s molecular cascades of flexibility, learning, and memory [54]. Insulin has a wide range of effects on the CNS and regulates critical processes such as energy homeostasis, endocrine reproduction, learning, memory, and neuronal survival in adults [5]. Insulin regulates neural proliferation, apoptosis, and synaptic transmission, and has pleiotropic effects on neurons [55]. There is a mechanical link between Aβ metabolism and insulin resistance. It has been shown that in advanced AD cases, the higher the Aβ level, the more insulin receptors are removed from the cell surface [52]. Studies have shown that IR or deficiency impairs learning and memory, so that insulin administration improves working memory and cognition and increases Aβ42 clearance in the brain [56].

Alzheimer’s disease should be considered as a degenerative metabolic disease due to brain insulin resistance and deficiency. Impaired insulin signaling can involve many essential abnormalities in AD, including neuroinflammation [57]. Neuroinflammation exacerbates IR, neurotoxicity, and cell death due to oxidation, gliosis, and toxicity of Aβ42 means that the association between IR and neuroinflammation is very close [57].

### Inflammation and obesity

PCOS women are at higher risk for impaired fat metabolism. Abdominal obesity in PCOS patients is related to IR, hyperandrogenism, regular ovulation, and inflammation [40, 41]. On the other hand, central obesity exacerbates endocrine and metabolic disorders in PCOS [58]. Obesity is associated with an increase in inflammatory factors; including interleukins and peptides associated with the calcitonin gene, and it can upregulate ovarian androgen production [59]. Inflammatory genes, such as interleukin-1 beta (IL-1β), IL-8, leukemia inhibitory factor (LIF), NOS2, and prostaglandin-endoperoxide synthase 2 (PTGS2), are over-expressed in granulosa cells (GCs) of PCOS patients, indicating inflammation of the ovarian GC responses [60]. TNFα, as a proinflammatory agent, may exacerbate the development of IR in PCOS women [3].

Neuro-inflammation is a primary and consistent feature in many neurodegenerative diseases, including AD [61]. Overweight people and diabetics are at higher risk for cognitive impairment and dementia [45], and by comparing obese and thin patients of the same age, obese patients show a higher degree of hyperandrogenism. Some studies have also shown that free fatty acids promote the development of amyloid fibers and tau in vitro [62], and therefore, obesity is associated with mild cognitive impairment and changes in the structure and function of the hippocampus [63, 64]. Free fatty acids contribute to Alzheimer’s pathology by causing inflammation, enhancing Aβ deposition, and inhibiting clearance of Aβ [65].

Increased expression of proinflammatory cytokines in the vicinity of Aβ42 plaques indicates that neuroinflammation is an essential mediator of AD neuro-degradation [66].

Also, neuro-inflammation promotes neuronal injury and cholinergic dysfunction [67]. In this regard, epidemiological studies have shown that people who take chronic anti-inflammatory drugs or antioxidants have a lower risk of developing cognitive impairment and AD [68]. Increased systemic inflammation is thought to cause changes in the microglia, resulting in inflammation in the CNS, and may increase the risk of cognitive aging and Alzheimer’s disease [69].

Because sleep disorders have essential effects on inflammatory biology, inflammation may be a biologically acceptable pathway and a link between sleep disorders and the risk of Alzheimer’s disease [70].

### Obstructive sleep apnea (OSA)

Obstructive sleep apnea (OSA) is characterized by partial or total obstruction of the upper airways and recurrence during sleep that leads to intermittent hypoxemia, of which obesity is a pillar of its physical pathology [71]. Obese women with PCOS are more likely to develop obstructive sleep apnea than healthy women [72, 73].

Studies show that androgens affect sleep patterns and lead to OSA development [74, 75]. As a result, women with PCOS have higher respiratory sleep levels, which may be associated with increased androgen levels associated with the syndrome [71]. Due to hormonal disorders, the prevalence of OSA is higher in women with PCOS compared with women without PCOS [76]. A wide range of hormonal and metabolic abnormalities in PCOS is also associated with people's hormonal profiles with OSA [77]. The increasing prevalence of obstructive sleep apnea in PCOS patients is associated with an increase in androgens or a decrease in estrogens and an increase in visceral adiposity [78]. Studies have shown that higher levels of testosterone in PCOS patients are related to OSA [79]. This is why sleep disorders in patients with PCOS are twice as common as in ordinary people [80]. Hyperandrogenism, IR, and low estrogen and progesterone levels with PCOS have all been suggested to cause OSA in PCOS patients [81]. IR is associated with irregular sleep breathing [3]. Progesterone increases respiratory pressure and the muscles’ function that dilates the upper airway [82]. This is why hormone therapy in postmenopausal women can be a protective factor against obstructive sleep apnea syndrome.

Evidence suggests that sleep disorders may lead to cognitive decline by increasing β-amyloid load, which increases the risk of dementia in AD [15]. On the other hand, respiratory disorders during sleep increase the risk of dementia and AD [83]. In humans, the concentration of β-amyloid in CSF fluctuates and increases during the day and decreases at night, and sleep deprivation or insomnia can affect the pattern of secretion [84].

Animal studies show that sleep increases clearance of soluble β-amyloid, and sleep–wake activity disorders interfere with eliminating potentially neurotoxic waste products, such as β-amyloid, which accumulate during the waking period [85, 86]. Evidence suggests that depression is a risk factor for cognitive decline and dementia and that sleep disorders and insomnia can be risk factors for depression and recurrence of depression [87, 88]. As a result, it can be said that there is usually a two-way relationship between sleep disorders and depression, also between sleep disorders and dementia.

Sleep is recognized as an essential modulator of several aspects of endocrine function, making it challenging to elucidate the relationship between these factors [76]. However, there is no evidence that treatment for sleep disorders prevents cognitive decline or dementia.

### Vitamin D

Over the past 25 years, vitamin D has been recognized as a serious candidate for the nervous system’s development and functioning and a treatment option for several neurological pathologies [89].

Researchers have recently shown that vitamin D deficiency is common in PCOS and that vitamin D levels are associated with reproductive ability, metabolic changes, and mental health in PCOS patients [90]. In this regard, Krul-Poel et al. showed that women with PCOS and infertility have lower serum levels of 25 (OH) D than the fertile control group [91].

Vitamin D deficiency in PCOS may lead to IR [38], so vitamin D concentration is negatively correlated with IR parameters and body fat mass. Also, treatment with vitamin D improves the metabolism of IR and lipids, improving the metabolic disorders of PCOS patients [90].

Vitamin D increases the maturation of adipose cells, activates enzymes involved in lipid and carbohydrate metabolism, and increases adipose tissue [92].

Vitamin D levels are inversely related to serum androgen levels. Vitamin D administration lowers serum androgen and anti-Müllerian hormone (AMH) levels and reduces endometrial thickness; this reduction in androgen levels in PCOS patients improves the menstrual cycle folliculogenesis [90].

Studies show that vitamin D deficiency is associated with the onset of the first symptoms of AD and can contribute to the onset of dementia. However, interventional studies did not improve cognitive function after administering vitamin D supplementation [89]. Vitamin D plays an essential role in metabolic pathways, including calcium homeostasis, the insulin pathway, and the synthesis of sex hormones, all of which are affected by PCOS [93].

The main reason for taking vitamin D supplementation in PCOS women is its role in suppressing proinflammatory cytokines, glucose metabolism, increasing insulin receptor expression, plus synthesis and secretion of insulin [94]. Vitamin D deficiency is associated with many human diseases, especially age-related diseases, such as AD, cardiovascular disease, cancer, type II diabetes, multiple sclerosis, and various inflammatory disorders [95]. The progression of dementia and non-communicable diseases such as AD is a major public health problem. One of the direct causes of neuronal loss and decreased recognition of Aβ accumulation is associated with increased inflammatory responses in the brain [96]. Vitamin D deficiency is a growing problem and plays a significant role in the cytotoxicity of amyloid plaques, which affect a significant portion of the population in many countries [97]. Vitamin D is involved in genomic and non-genomic effects on calcium homeostasis, neurotransmission, oxidative stress, Aβ and Tau accumulation, vascularization, and inflammation; all of these pathways can be impaired in AD [98]. In addition to calcium homeostasis, vitamin D has numerous functions in the nervous system, including regulating the production of neurotrophic factors, neurotransmitter secretion, oxidative stress mechanisms, modulating the immune system, and is currently known as an effective immune modulator [89]. It also has the potential to regulate the inflammatory status in AD pathology [89].

### Hyperandrogenism

Androgens belong to the family of steroid hormones and hyperandrogenism is one of the main clinical manifestations of PCOS [99]. Androgens include testosterone, androstenedione (A4), dihydrotestosterone (DHT), dehydroepiandrosterone (DHEA), and dehydroepiandrosterone sulfate (DHEAS) [100]. Serum levels of various androgens in PCOS patients are constantly increasing compared to healthy individuals [101]. Approximately 75% of PCOS patients having hyperandrogenism, and more than 80% having abnormally free testosterone levels [102]. This may be due to oxidative stress and hyperinsulinemia, which stimulate theca-interstitial cell proliferation and androgen production [103, 104]. Studies have shown that exposure of female fetuses to androgen overload in all models causes PCOS characteristics [105].

On the other hand, androgens may help reduce the process of AD, and decreased levels of gonadal hormones, especially estrogens and androgens, are commonly associated with the onset of AD [42]. Some studies have shown that androgens’ administration improves cognitive function [59]; androgen administration reduces the expression of inflammatory factor IL-1β, so reduces nerve death in the hippocampus [106].

Sex hormone-binding globulin (SHBG) is a sex steroid produced in the hepatic cells, and its low production may be involved in the pathogenesis of PCOS [107]. SHBG has a slight tendency to bind estradiol and a high affinity for testosterone [108]. The biological activity of androgens is also determined by free testosterone, and therefore investigation of SHBG levels is essential in assessing hyperandrogenism [107]; in PCOS patients, one of the reasons for low serum SHBG levels is hyperandrogenemia [109]. SHBG binds to free androgens and lowers free androgen levels, then reducing hyperandrogenism and IR [109]. SHBG levels increase in AD patients and lower serum levels of bioactive sex steroids [21].

## Discussion

The HPG axis plays an important role in the pathophysiology of PCOS, which is one of the most common endocrine and metabolic disorders [110]. Hormones, especially gonadal hormones, widely affect the function and stability of the central nervous system [111]. Also, cognitive function may depend on the level of sex steroids and gonadotropins, including HPG-based dysfunction [21].

This study investigated some common factors such as IR, inflammation, sleep apnea, vitamin D deficiency, LH/FSH ratio, and sex hormones that may play a role in the association between AD and PCOS.

Four phenotypes have been defined for PCOS (According to Rotterdam criteria), including complete PCOS with hyperandrogenism, ovulation disorder, and polycystic ovaries; PCOS with hyperandrogenism and ovulation disorders; PCOS with hyperandrogenism and polycystic ovaries; PCOS associated with ovulatory and polycystic ovary disorders [112]. Studies have shown that the incidence of the disease among PCOS patients is significantly different based on ethnicity, phenotype, and morbidity. It seems that the component of hyperandrogenism is the most important determining factor in the pathophysiology of PCOS among other factors and also this factor is the main predictor of metabolic disorders observed in the disease [113]. It is important to note that the major determinants of the pathophysiology of PCOS may not necessarily be the determinants of the vulnerability of these patients to neurodegenerative Alzheimer’s disease. Evidence-based studies should be performed to determine which phenotype of PCOS leads to Alzheimer’s disease.

Obesity and IR, which are also seen in PCOS patients, may increase the risk of cognitive decline and neurodegenerative diseases [53]. On the other hand, due to the increased levels of androgens in PCOS patients, we expect cognitive activity to increase in these patients. However, oral contraceptive pills (OCPs) are drugs prescribed to women with PCOS as a first-line treatment to reduce androgen overexpression and regulate menstrual cycles [114]. Because OCPs reduce androgens, they can have adverse effects on memory and cognition. Also, drugs such as *Metformin*, which reverses IR, are associated with increased SHBG levels in women with PCOS [115]. As mentioned above, SHBG levels also increase in patients with AD. The use of these drugs may increase the symptoms of AD by increasing the level of SHBG. Contradictory results have been reported on the effects of metformin on central nervous system function and pathology. Some studies have shown increased neurogenesis, improved spatial learning, and decreased cognitive impairment, but others have shown negative consequences such as the increased risk of AD. Cognitive dysfunction has been shown to impair neuronal viability in the AD mouse model. There is also evidence that metformin reduces the activity of acetylcholinesterase (AChE), which is responsible for the breakdown of acetylcholine (Ach), a neurotransmitter involved in learning and memory. Therefore, the effect of metformin on Alzheimer’s disease is multifaceted [116, 117]. Although more research is needed, controlling SHBG levels may keep endogenous estrogens and androgens bioactive and improve cognition. Due to the variety of actions of estrogen, LH, and testosterone, combination hormone therapy may be more effective in preventing AD. Of course, the negative effects of hormone therapy in different reproductive periods of women before and after menopause should not be ignored [118].

Studies in this area are not adequate, so more animal research and studies in human research such as case–control studies may also help to give more insights into the potential association between PCOS and AD.

## Conclusion

In general, the results of our study identified the following highlights:

- Accumulation of amyloid-beta and tau proteins is a prominent feature of Alzheimer’s disease pathology. Increased levels of LH to FSH are involved in the metabolism and accumulation of Aβ and reduce BDNF in the brain.
- Calcium signaling by vitamin D in neurons is crucial for neurotransmission and maintenance of synaptic plasticity and LTP.
- Insulin signaling also affects hippocampal plasticity, learning, and memory.
- Free fatty acids increase inflammation by causing Aβ deposition and resulting in nerve damage.
- In patients with PCOS, increased LH to FSH ratio, decreased vitamin D, insulin resistance, and obesity are some of the most important factors that may increase the risk of Alzheimer’s disease.

## Data Availability

All data generated or analyzed during this study are included in this published article.

## Abbreviations

PCOS: Polycystic ovary syndrome
AD: Alzheimer disease
IR: Insulin resistance
HA: Hyperandrogenism
CNS: The central nervous system
Aβ: Amyloid beta-peptide
APP: Amyloid precursor protein
HPG: Hypothalamic–pituitary–gonadal
ERs: Estrogen receptors
BDNF: Brain-derived neurotrophic factor
GnRH: Gonadotropin-releasing hormone
IL-1β: Interleukin-1 beta
PTGS2: Prostaglandin-endoperoxide synthase 2
LIF: Leukemia inhibitory factor
TNFα: Tumor necrosis factor-alpha
OSA: Obstructive sleep apnea
AMH: Anti-Müllerian hormone
LH: Luteinizing hormone
FSH: Follicle-stimulating hormone
LHR: Luteinizing hormone receptors
DHT: Dihydrotestosterone
DHEAs: Dehydroepiandrosterone sulfate
OCPs: Oral contraceptive pills
SHBG: Sex hormone-binding globulin
